# The mitochondrial *ATPase6* gene is more susceptible to mutation than the *ATPase8* gene in breast cancer patients

**DOI:** 10.1186/1475-2867-14-21

**Published:** 2014-03-03

**Authors:** Massoud Ghaffarpour, Reza Mahdian, Forouzandeh Fereidooni, Behnam Kamalidehghan, Nasrin Moazami, Massoud Houshmand

**Affiliations:** 1 Medical Genetics Department, National Institute for Genetic Engineering & Biotechnology, Tehran, Iran; 2 Biotechnology Research Center, Molecular Medicine Department, Pasteur Institute of Iran, Tehran, Iran; 3 National Cancer Institute, Imam Khomeini Hospitals Complex, Tehran University of Medical Science, Tehran, Iran; 4 Pharmacy Department, Faculty of Medicine, University of Malaya, Kuala Lumpur, Malaysia; 5 Iranian Research Organization for Science and Technology, Tehran, Iran

**Keywords:** MtDNA, *ATPase6*, *ATPase8*, Breast cancer

## Abstract

**Background:**

Breast cancer is the most common malignancy in women throughout the world. Mitochondria play important roles in cellular energy production, free radical generation and apoptosis. Identification of mitochondrial DNA mutations and/or polymorphisms as cancer biomarkers is rapidly developing in molecular oncology research.

**Methods:**

In this study, the DNA alterations of the mitochondrial *ATPase 6* and *8* genes were investigated in 49 breast cancer patients using PCR amplification and direct DNA sequencing on mtDNA. A possible association between these variants and tumorigenesis was assessed. Furthermore, the impact of non-synonymous substitutions on the amino acid sequence was evaluated using the PolyPhen-2 software.

**Results:**

Twenty eight distinct somatic mitochondrial DNA variants were detected in tumor tissues but not in the corresponding adjacent non-tumor tissues. Among these variants, 9 were observed for the first time in breast cancer patients. The mtDNA variants of A8384 (T7A), T8567C (I14T), G8572A (G16S), A9041G (H172R) and G9055A (A177T) showed the most significant effects probably due to damaging changes to the resulting protein. Furthermore, non-synonymous amino acid changing variants were more frequent in the *ATPase6* gene compared to the *ATPase8* gene.

**Conclusion:**

Our results showed that the *ATPase6* gene is more susceptible to variations in breast cancer and may play an important role in tumorigenesis by changing the energy metabolism level in cancer cells.

## Introduction

Breast cancer is a major public health problem in women throughout the world. In 2008, it was the most common cause of death in women (458,000 deaths) worldwide [1]. Breast cancer is also the most common cancer and the fifth cause of mortality due to malignancies among Iranian women [1,2].

The human mitochondrial genome consists of a circular double-stranded DNA of 16,569 base pairs, including genes encoding for the electron transport chain (complexes I-IV), ATP synthase or complex V in oxidative phosphorylation as well as a displacement loop region, 2 ribosomal RNAs (16 and 23) and 22 transfer RNAs [3,4]. Variants of *ATPase subunit 6* (8366–8572) and *ATPase subunit 8* (8527–9207) in mitochondrial DNA (mtDNA) has been reported in different types of cancers, including breast, colon and ovarian [5-7].

The mitochondrion plays a critical role in cellular energy production [8], carcinogenesis and tumor progression, and could be a prognostic marker in different cancer types [5,9-15]. To date, various types of mtDNA alterations, including point variants, large deletion and copy number changes have been reported in breast, colon and ovarian cancers [5,16]. There is strong evidence that mtDNA alterations can enhance oxidative stress and the risk of tumor development as well as tumor initiation, proliferation [17], metastasis [18-20] and resistance of cancer cells to apoptosis [21].

Therefore, this study was undertaken to evaluate mitochondrial *ATPase6* and *8* alterations in tumor and adjacent non-tumor tissues in breast cancer patients. We also investigated the correlation between the variants in these genes and the clinico-pathological features in these breast cancer patients.

## Materials and Methods

### Tumor tissue collection

Forty-nine breast cancer patients (34–75 years of age with a median age of 52.43 years) took part in this study. The patients were referred to the National Cancer Institute (NCI) at Imam Khomeini Hospital Complex, Tehran, Iran, from Oct. 2007 to Oct. 2009. Tumor tissue and adjacent non-tumor tissue samples were obtained from the Iranian National Tumor Bank (INTB) at NCI. Each specimen was immediately frozen following resection and stored at −80°C until DNA extraction. The pathologic changes in tumor samples were confirmed by two expert pathologists as adenocarcinomas according to the American Joint Committee on Cancer [22]. None of the patients received chemotherapy or radiotherapy treatment before they underwent surgery. All patients were informed on the aim of the study and signed an informed consent approved by the INTB Ethical Committee for the genetic analysis.

### DNA extraction and PCR

In order to identify the alterations in the mtDNA *ATPase6* and *ATPase8* genes, PCR-sequencing was performed as described previously with some modifications [23] Total genomic DNA was extracted from fresh tumor samples containing at least 90% neoplastic cells, as well as their adjacent non-tumor tissues, using the QIAamp Mini Kit (USA). The sequences of the primers were as follows: F-ATPase: 5′- CTACGGTCAATGCTCTGAAA -3′ (Accession No. NC_012920.1, 8161–8180). R-ATPase: 5′-TACTATATGATAGGCATGTGA-3′ (9219–9239). PCR amplification was performed using a ready-to-use PCR master mix (Sinaclon LTD, Tehran, Iran) in a final volume of 50 μl containing 5 ng of genomic DNA and 0.10 μM of each primer in a MJ Mini Gradient Thermal Cycler PTC-1148 (Bio-Rad, USA). PCR amplification was carried out with the following program: a 5-min pre-PCR incubation step at 95°C, 35 cycles of 95°C for 60 s, annealing temperature at 55°C for 1 min and 72°C for 2 min, and a final extension of 72°C for 10 min. The amplified fragment (1078 bp) was observed on 1.5% agarose gel.

### Sequencing analysis

The PCR products were sequenced using the previously reported primers [23] on a ABI Prism 3700 automated sequencer (Applied Biosystems, USA). Sequence analysis was carried out using the FinchTV 1.4 software (Geospiza, Inc., USA). The sequences were compared to the human mtDNA reference sequence (Gene Bank ID: NC_012920.1) using the BLAST sequence analysis tool (NCBI, Bethesda, USA). The Mitomap database was used to identify mitochondrial genome sequence variants.

### Prediction of pathogenicity by protein modeling analysis

The impact of non-synonymous (coding) substitutions in the resulting protein was assessed using PolyPhen-2 (v. 2.2.2) software, a tool for predicting the possible impact of an amino acid substitution variant on the structure and function of the corresponding protein, which is interpreted as benign and damaging effects [24].

### Statistical analysis

The correlation between each alteration in the *ATPase6* and *ATPase8* genes in tumor samples and their adjacent normal tissue were analyzed by Fisher’s exact test using statistical package SPSS (v.16.1). The correlation between the groups was considered statistically significant if the *p-*value was less than 0.05. Additionally, for each variant the odds ratio (OR) and 95% confidence interval (95% CI) were calculated in order to determine its association to the increased risk in breast cancer patients. The association between mtDNA alteration and clinico-pathological characteristics of breast cancer patients with more than one missense mutation was evaluated using One-way ANOVA analysis.

## Results

In this study, the complete sequences of the *ATPase6* and *8* genes of 49 tumor tissues and adjacent non-tumor tissues were analyzed in a cohort of breast cancer patients. The clinico-pathological characteristics of the patients are summarized in Table 1. From 49 breast cancer cases, 28 mtDNA variants were found in tumor tissues, which were not present in their adjacent normal tissues. From 28 variants, 23 (82.14%) were found in the *ATPase6* gene and the remaining 5 sequence variants were detected in the *ATPase8* gene. All cases showed variants in the *ATPase6* gene, whereas only 8.16% (4 of 49) cases had variants in the *ATPase8* gene. Among 28 mtDNA alterations, 26 were at the homoplasmic state and the remaining 2 variants were at the heteroplasmic state (Table 2). However, there was no significant correlation (*P* > 0.05) between the *ATPase6* and *8* gene variants and the clinico-pathological characteristics of the patients (Table 1). Our results indicated that the A8860G variant was detected in 100% of tumor tissue samples compared to adjacent non-tumor tissues, showing that this alteration may significantly increase breast cancer risk (*P* < 0.05). However, the patients’ survival was shorter in cases with more than one mtDNA non-synomous *ATPase* variant compared to the patients with only one mtDNA non-synonymous *ATPase* variant (A8860G ) (*p* =0.051, Table 1).

**Table 1:** **Characterization of clinico-pathological parameters and the frequency of cases with more than one somatic mtDNA (*ATPase6/8 *) mutation in breast cancer patients**

**Frequency of patients in each group**		**Patients with more than one somatic mtDNA ( *ATPase6/8 *) mutation**		
**Variable**	**n (%)**	**n (%)**	OR; (95% CI)*	**P value**
**Total number of patients**	49			
**Age at diagnosis (Yrs)**			1.482(0.403-5.451)	0.746
<50	19(42.2)	5(26.3)		
≥50	26(57.8)	9(34.3)		
**Histological grade**				0.121
I	13(29.5)	1(7.7)		
II	24 (54.5)	9(37.5)		
III	7(15.9)	3(42.9)		
**TNM(AJCC) stage**				0.680
I	3(6.7)	1(33.3)		
II	10(22.2)	2(20.)		
III	3(6.7)	0(0)		
IV	(64.4)29	11(37.9)		
**Tumor size(cm)**				0.889
<2	5(11.1)	2(40)		
2-5	30(66.7)	9(30)		
>5	10(22.2)	3(30)		
**Lymph node status**			1.176(0.281-4.926)	1.000
Negative	12(30.8)	4(33.3)		
Positive	27(69.2)	10(37)		
**Lymphatic invasion**			1.077(0.281-4.127)	1.000
Negative	18(47.4)	6(33.3)		
Positive	20(52.6)	7(35)		
**Vascular invasion**			2.292(0.511-10.284)	1.000
Negative	13(32.5)	4(30.8)		
Positive	27(67.5)	10(37)		
**Estrogen receptor status**			0.357(0.075-1.704)	0.222
Negative	8(17.4)	4(50)		
Positive	38(82.6)	10(26.3)		
**Progesterone receptorstatus**			0.938(0.265-3.313)	1.000
Negative	22(48.9)	7(31.8)		
Positive	23 (51.1)	7(30.4)		
**Her-2/neu receptor**			1.061(0.285-3.948)	1.000
Negative	30(65.2)	9(30)		
Positive	16(34.2)	5(31.3)		
**P53**			0.625(0.166-2.356)	0.526
Negative	23(53.50)	8(34.8)		
Positive	20(46.5)	5(25)		
**Cancer metastasis**			3.056(0.718-13.011)	0.191
Negative	18(38.3)	3(16.7)		
Positive	29(61.7)	11(37.9)		
**Overall survival (5 yr%)**	18 of 41(43.9)	3(16.7)	0.218(0.049-0.963)	0.051

^*^OR; Odds ratio, (95% CI); confidence interval reflects a significance level of 0.05.

**Table 2:** **Frequency of mtDNA *ATPase 6/8 *** **gene sequence alterations in 49 breast cancer patients**

**No**	**Locus**	**Allele**	**Nucleotide position**	**Nucleotide change**	Amino acid change*	Mutation status**	**Frequency**	OR; 95% CI^***^	***P Value***	**Reference**
1	MT-ATPase8	A8384G	8384	A-G	**T7A**	Hm	1	1.021;0.980-1.063	0.315	NR^****^
2	MT-ATPase6	T8542C	8542	T-C	**F6L**	Hm	1	1.021;0.980-1.063	0.315	NR
3	MT-ATPase8	T8542C	8542	T-C	**C59C**	Hm	1	1.021; 0.980-1.063	0.315	NR
4	MT-ATPase6	G8557A	8557	G-A	**A11T**	Hm	1	1.021; 0.980-1.063	0.315	Colonic crypts cancer [34], Breast cancer [27,28]
5	MT-ATPas8	G8557A	8557	G-A	L64L	Hm	1	1.021; 0.980-1.063	0.315	Alzheimer's disease [40]
6	MT-ATPase6	T8567C	8567	T-C	**I14T**	Hm	1	1.021; 0.980-1.063	0.315	Parkinson's disease [42]
7	MT-ATPas8	T8567C	8567	T-C	**S68P**	Hm	1	OR 1.021;: 0.980-1.063	0.315	Parkinson's disease [49]
8	MT-ATPase6	G8572A	8572	G-A	**G16S**	Hm	1	OR 1.021; 0.980-1.063	0.315	Thyroid tumor [50]
9	MT-ATPas8	G8572A	8572	G-A	**G69S**	Hm	1	1.021; 0.980-1.063	0.315	Colonic crypts cancer [34]
10	MT-ATPase6	C8684T	8684	C-T	**T53I**	Hm	1	1.021; 0.980-1.063	0.315	Multiple Sclerosis [51], Ataxia telangiectasia [21], Huntington [52], Autism [53], Osteosarcoma [54],
11	MT-ATPase6	T8697C	8697	T-C	**I24T**	Hm	1	1.021; 0.980-1.063	0.315	Thyroid tumor [50], Multiple Sclerosis [51], Ataxia telangiectasia [21], Breast cancer [30], Colorectal adenomatous polyps [40]
12	MT-ATPase6	A8701G	8701	A-G	**T59A**	Hm	2	1.043; 0.984-1.105	0.153	Thyroid tumor [50], Ataxia telangiectasia [21], Breast cancer [27,29], colorectal adenomatous polyps [38], Osteosarcoma [54]
13	MT-ATPase6	T8777C	8777	T-C	F117F	Hm	1	1.021; 0.980-1.063	0.315	NR
14	MT-ATPase6	C8794T	8794	C-T	**H90Y**	Hm	2	1.043; 0.984-1.105	0.153	Exercise Endurance/Coronary Atherosclerosis risk[32]
15	MT-ATPase6	A8860G	8860	A-G	**T112A**	Hm	49		0.000	Colorectal cancer [36,38], Ovarian cancer [37], Breast cancer [27,29,34], Human glioma cells [33], Osteosarcoma [54], Leber's hereditary optic neuropathy [35]
16	MT-ATPase6	T8877C	8877	T-C	F117F	Hm	3	1.065; 0.992–1.114	0.079	Leber's hereditary optic neuropathy [55]
17	MT-ATPase6	T8881C	8881	T-C	**S119P**	Ht	1	1.021; 0.980-1.063	0.315	NR
18	MT-ATPase6	C8910T	8910	C-T	F128F	Ht	2	1.043; 95% CI: 0.984-1.105	0.153	The southern belt of Siberia population [56]
19	MT-ATPase6	G8950A	8950	G-A	**V142I**	Hm	2	1.043; 0.984-1.105	0.153	Huntington [54],LDYT [57]
20	MT-ATPase6	G8994A	8994	G-A	L156L	Hm	1	1.021; 0.980-1.063	0.315	Ataxia telangiectasia [21], Breast cancer [27], Colorectal adenomatous polyps [38]
21	MT-ATPase6	C9003A	9003	C-A	R159R	Hm	1	OR 1.021; 0.980-1.063	0.315	NR
22	MT-ATPase6	A9007G	9007	A-G	**T161A**	Hm	1	1.021; 0.980-1.063	0.315	Deafness associated [58]
23	MT-ATPase6	A9041G	9041	A-G	**H172R**	Hm	1	1.021; 0.980-1.063	0.315	NR
24	MT-ATPase6	G9055A	9055	G-A	**A177T**	Hm	3	1.065; 0.992–1.114	0.079	Colorectal cancer [36], Colorectal adenomatous polyps [38], Breast cancer [28,30], Non-muscle invasive bladder cancer [44], Osteosarcoma [54], Pancreatic cancer [43], Parkinson's disease protective factor [45]
25	MT-ATPase6	G9085A	9085	C-T	**P187S**	Hm	1	1.021; 0.980-1.063	0.315	NR
26	MT-ATPase6	T9090C	9090	T-C	S188S	Hm	1	1.021; 0.980-1.063	0.315	Colorectal cancer [59] Leber's hereditary optic neuropathy [60]
27	MT-ATPase6	T9148C	9148	T-C	L208L	Hm	1	1.021; 0.980-1.063	0.315	Occipital stroke [61]
28	MT-ATPase6	C9168T	9168	C-T	F214F	Hm	1	1.021; 0.980-1.063	0.315	NR

Abbreviations:

*Missense mutations are in bold.

**Hm: Homoplasmic, Ht: Heteroplasmic.

*** OR; Odds ratio, (95% CI); confidence interval reflects a significance level of 0.05.

****NR; Not reported in mitomap website.

Furthermore, the damaging impact of an amino acid substitution on the structure and function of the ATPase6 and 8 proteins was predicted using PolyPhen-2 software (Table 3). The mtDNA variants A8384 (T7A), T8567C (I14T), G8572A (G16S), A9041G (H172R) and G9055A (A177T) showed significant effects on the resulting protein. However, there was no significant association between mtDNA alterations and the clinico-pathological characteristics of breast cancer patients.

**Table 3:** Impact of non-synonymous* (coding) substitutions on *the ATPase6 * **and *8 *** **genes**

**Non-synonymous coding substitutions**	**Damaging score**	**Benign score**
***ATPase 6*** **gene**		
**T8542C( F6L)**	0.976	0.917
G8557A (A11T)	0.002	0.004
T8567C (I14T)	0.617	0.280
G8572A (G16S)	0.895	0.498
C8684T (T53I)	0.005	0.005
A8701G (T59A)	0.002	0.005
C8794T (H90Y)	0.002	0.003
A8860G (T112A)	0.000	0.003
**T8881C (S119P)**	0.325	0.149
G8950A (V142I)	0.000	0.001
A9007G (T161A)	0.994	0.988
**A9041G H(172R)**	0.854	0.331
G9055A (A177T)	0.854	0.331
***ATPase 8*** **gene**		
A8384G (T7A)	0.845	0.399
T8542C (S68P)	0.000	0.000

Non-synonymous variants were predicted as damaging and benign (With a score of 0 to 1) based on effects on the resulting protein using PolyPhen-2 software.

The new variants are in bold format.

## Discussion

The identification of mitochondrial DNA mutations and/or polymorphism patterns is rapidly developing in the field of molecular oncology. A large number of somatic mutations in the mitochondrial genome have been recently reported in different types of cancer,luding breast, colon and ovarian cancers [5,6]. These molecular markers may have potential implication in cancer research.

Mitochondrial complex V genes play an important role in ATP production [25] and the apoptosis pathways [5]. The contribution of mtDNA complex V variants in cell transformation, elevated ROS production, and tumor progression has been described previously [26]. Moreover, efficient programmed cell death needs the molecular machinery of ATP synthase [27].

The *ATPase6* gene, one of the complex V genes, contributes to mtDNA maintenance [25]. Furthermore, the *ATPase8* variants have been detected in rat and human bladder cancer cells developed through chemically-induced carcinogenesis [28]. In a meta-analysis study carried out by Lu et al. a total of 55 variants, comprising 34 missense variants, 20 silent variants and 1 nonsense variant, were found in the *ATPase6* gene and a total of 9 variants, including 2 missense variants and 7 silent variants, were detected in the *ATPase8* gene [6].

In our study, among 28 distinct somatic variants, 18 were missense variants. Six variants have been previously reported in breast cancer [29-32] and 9 variants were new, including 4 missense and 5 silent variants which were observed for the first time in breast cancer patients. However, 17 variants were previously reported in other types of cancers and diseases (Table 2). In addition, more non-synonymous amino acid changing variants were found in the *ATPase6* gene in comparison with the *ATPase8* gene (Table 2). Our findings suggest that in breast cancer patients, the *ATPase6* gene might be more susceptible to mutation in comparison to the *ATPase8* gene. Shidara et al. and Kirches reported that *ATPase6* gene variants may enhance cancer progression by preventing apoptosis pathways [6,33].

The functional role of *ATPase6/8* variants in tumorigenesis is debatable; however, some of these variants are located in structurally and functionally important regions of the proteins. For instance, the A8860G alteration in *ATPase6* has been reported as a polymorphism in different studies [29,31,34-40]. The frequency of this polymorphism has been reported to be from 79–91.66% in breast cancer patients [30,31], 75-100% in other types of cancers [38-40] and 92.85%-100% in neurodegenerative diseases [37,41-43]. Our results indicated that the A8860G variant was present in 100% of tumor tissue samples. Although this variant is located in a poorly conserved protein region with no impact on protein structure based on PolyPhen-2 software (Table 3), the variation may still contribute to other mtDNA and nDNA mutations.

The frequency of the G9055A variation has been reported as either 10.5% [28] or 18.6% [30] in breast cancer patients, indicating that it may increase the risk of breast cancer progression (OR: 3.03, 95% CI: 1.63-5.63, *P* = 0.0004) [32,44]. This variation is located in a conserved protein region with damaging impact on protein structure (Table 3). Furthermore, the frequency of this polymorphism has been reported as 10% in pancreatic cancer [45] and as 57% and 100% in tubular and villous adenomas, respectively [40]. Moreover, the high frequency of this variation has been shown in non-muscle invasive bladder cancer [46]. In addition, this polymorphism has been reported as a protective factor (OR: 0.46, 95% CI: 0.22-0.91, *P* = 0.03) in Caucasian women with Parkinson’s disease [47]. From these results, we propose that this mtDNA variation is unfavorable for neurodegenerative disorders, while having a protective effect on cancer. According to our results, the frequency of this variation was 6.12% (3 of 49) in tumor samples.

A study by Petros et al. indicated that T8993G in *ATPase6* can contribute to tumor growth in nude mice [48]. Another study showed that cybrids with a T8993G or T9176 *ATPase6* mutation in nude mice can contribute to tumor development by preventing apoptosis in the early stages of tumor growth [10]. However, we detected none of these mutations in breast cancer patients.

Based on our results, the existence of more than one missense variants in some cases with different clinico-pathological features (Table 4) suggests a synergistic effect of different mtDNA variations on carcinogenesis.

**Table 4:** MtDNA alterations and clinico-pathological characteristics of breast cancer patients with more than one missense mutation

**Case**	**Locus**	**Variant**	**Frequency**	**Age (Yrs)**	**Grade**	**Tumor size (cm)**	TNM*	**Stage**
BC-6	*ATPase6*	A8384G	4	44	III	3	T2N1M0	II
		T8542C						
	*ATPase8*	T8542C						
		A8860G						
BC-10	*ATPase6*	A8860G	3	55	III	2.5	T2N0M1	IV
		G8950A						
		A9041G						
BC-19	*ATPase6*	A8860G	2	42	II	5	T3N2M1	IV
		G9055A						
BC-20	*ATPase6*	A8860G	2	68	III	1.8	T2N1M1	IV
		A9007G						
BC-21	*ATPase6*	A8860G	2	43		1.2	T1NXM1	IV
		G8950A						
BC-23	*ATPase6*	A8860G	2	36	III	10	T3N3M1	IV
		G9055A						
BC-25	*ATPase6*	A8860G	2	50	II	13	T4N3M1	IV
		C8794T						
BC-32	*ATPase6*	A8860G	2	74	I	5	T3N1M1	IV
		T8881C						
BC-35	*ATPase6*	C8794T	2	75	II	5	T3N3M1	IV
		A8860G						
BC-37	*ATPase6*	A8860G	2	67	II	2	T1N0M0	I
		G9095A						
BC-38	*ATPase6*	A8701G	3	69	II	3.5	T2N3M1	IV
		A8860G						
		T9085C						
BC-39	*ATPase6*	A8701G	2	59	III	3	T2N0M0	II
		A8860G						
BC-41	*ATPase6*	C8684T	2	51	II	3.5	T2N0M1	IV
		A8860G						
BC-48	*ATPase6*	T8567C	3	41	II	4.5	T2N3M1	IV
	*ATPase8*	T8567C						
		A8860G						

T1–T4: Size and/or extent of the primary tumor; NX: Regional lymph nodes cannot be evaluated; N0: No regional lymph node involvement (no cancer found in the lymph nodes); N1-N3:Involvement of regional lymph nodes (number and/or extent of spread); M0:No distant metastasis; M1:Distant metastasis (spread of cancer from one part of the body to another). There was no significant association between the mtDNA alterations and clinic-pathological characteristics of breast cancer patients.

In conclusion, the high frequency of *ATPase6* gene alterations in breast cancer proposes that mitochondrial gene variants may play an important role in tumorigenesis, changing the energy metabolism in cancer cells, and may be suggested as molecular biomarkers in breast cancer.

## Competing interests

The authors declare that they have no competing interests.

## Authors’ contributions

MG carried out the experimental procedures, participated in the sequence alignment and drafted the manuscript. RM, FF, and NM participated in the coordination of the study. BK wrote his constructive comments and edited the manuscript. MH conceived the project and supervised the study. All authors read and approved the final manuscript.
